# (Un)Broken: Lateral violence among hospital nurses, user violence, burnout, and general health: A structural equation modeling analysis

**DOI:** 10.3389/fmed.2022.1045574

**Published:** 2022-11-24

**Authors:** Maria Joao Vidal-Alves, David Pina, José Antonio Ruiz-Hernández, Esteban Puente-López, David Paniagua, Begoña Martínez-Jarreta

**Affiliations:** ^1^Department of Socio-Sanitary Sciences, University of Murcia, Murcia, Spain; ^2^Department of Community Medicine, Information and Health Decision Sciences, School of Medicine, University of Porto, Porto, Portugal; ^3^EPIUnit – Institute of Public Health, University of Porto, Porto, Portugal; ^4^Laboratory for Integrative and Translational Research in Population Health (ITR), University of Porto, Porto, Portugal; ^5^Department of Psychiatry and Social Psychology, University of Murcia, Murcia, Spain; ^6^Facultad de Derecho, Universidad Internacional de la Rioja (UNIR), Logroño, Spain; ^7^Department of Psychobiology and Methodology in Behavioral Sciences, Faculty of Psychology, Complutense University of Madrid, Madrid, Spain; ^8^Department of Pathological Anatomy, Forensic and Legal Medicine and Toxicology, University of Zaragoza, Zaragoza, Spain

**Keywords:** lateral violence, workplace, nursing, structural equation modeling, burnout, health, user violence

## Abstract

**Introduction:**

Workplace violence is a social problem yet to be solved. Although it is present in virtually all work environments, its prevalence in healthcare settings stands out, being perceived as something inherent to the job. Most studies in this context have focused on user violence against professionals. However, it has been observed that violence among colleagues in these types of jobs is a risk factor for the health of workers and has rarely been studied as a whole. Among the main consequences of exposure to violence reported in the literature, burnout syndrome, depression, anxiety, or somatic problems have been among the most studied. On the one hand, some authors claim that being exposed to workplace violence can increase the associated physical and psychological pathology and lead to a picture congruent with burnout. On the other hand, it has been hypothesized that violence is associated with burnout, which can trigger physical and psychological symptoms. Taking into account this background, the aim of this study is to explore workplace violence in health personnel, symptomatology, and burnout syndrome through mediation models that allow us to know the interrelationships between the variables.

**Methods:**

A cross-sectional design with a double descriptive-associative strategy was used. The sample was composed of 950 nursing professionals from public hospitals. The scales of physical and non-physical violence from users to professionals HABS-U, personal, social, and occupational violence among co-workers using the Health Aggressive Behavior Scale – Co-workers and Superiors (HABS-CS) scale, the burnout scale Maslach Burnout Inventory – General Survey (MBI-GS) which evaluates professional exhaustion, efficacy and cynicism, and the factors referring to depression, anxiety, somatization, and dysfunction of the GHQ-28 scale were applied. In order to calculate the models, workplace violence was used as a predictor of symptomatology, using the burnout variables as mediators. Regression coefficients with and without mediation model, direct and standardized estimates were obtained. For statistical power, Bootstrap analysis was used to calculate direct mediation effects.

**Results:**

After controlling the mediation effects of burnout and cynicism, physical and non-physical user violence toward healthcare personnel were significant predictors of the GHQ-28 scores. These same results were obtained when assessing the relationship between social, occupational, and personal violence among co-workers and GHQ-28 scores.

**Conclusion:**

Our results contribute to increase the evidence about the effects of violence on the health of professionals and to advance in the characterization of the possible consequent psychological damage. Regardless of the type of violence experienced, exposure to violence can lead to anxious, depressive or somatization symptoms, among others. Violence is also a predictor of burnout syndrome, which in turn accentuates the rest of the consequences studied. Despite the limitations of the proposed model, these results serve to highlight the complexity of the situation experienced by healthcare professionals. Moreover, it serves as a basis for proposing intervention/prevention programs to raise awareness and protect professionals from these risks. To this end, self-care tools should be proposed with which professionals take care of their own health through the management of violent situations and/or the improvement of occupational health.

## Introduction

Since violence is not always overt, a distinction is traditionally made between manifest and latent violence, meaning that the former is observable while the latter is not (1). When it is latent, a sense of unbalance emerges and impairs awareness, as happens with gaslighting, a form of psychological abuse that seeks to create doubt in a person, making them doubt their perceptions with the resource to persistent deception or mislead, or contradiction and lying, to disturb and affect the victim (2). Leymann, who studied trauma as a result of “psychological terror” in the workplace and unethical communication, used the term “mobbing” to describe this phenomenon (3, 4). In the workplace context, the terms mobbing and bullying are used in the literature as equivalent concepts, but they differ: while the former is used by a group of people who are not necessarily cruel or malicious against an individual, bullying is seen to involve a single aggressor (usually mean or vicious) who harasses alone, even when using supporters (5, 6). Authors also refer to the deliberate and reiterated nature of the acts as well as their severity, often using rumor, ostracism, silent treatment, humiliation, and intimidation of the target (7).

Workplace violence is described in Convention No. 190 of the International Labor Organization as “a range of unacceptable behaviors, practices or threats thereof, whether a single occurrence or repeated, that aim at, result in, or are likely to result in physical, psychological, sexual or economic harm and include gender-based violence and harassment.”

WHO’s ecological model of 2002 proposes a multifactorial perspective over workplace violence, encompassing biological, social, cultural, economic, and political interrelated factors [CITE (8)]. Chapel and DiMartino theorized on the multifaceted and interactive nature of workplace violence with a both institutional and personal reach, that has great applicability in healthcare context. It proposes that risk factors of violence with different natures interact and determine the outcomes for victims of violence and their organizations: contextual (e.g., globalization, increased vulnerability, and job insecurity), individual (e.g., age, sex, and personality), workplace (e.g., environmental and task-related), and societal (negative culture and violent society) depending also on who are the victims and perpetrators [CITE (9)].

For its consequences, workplace violence in healthcare (WPVH) has become a topic of great concern in many countries, making it clear that it has severe and long-lasting consequences (10–13) and requires good assessment in healthcare settings (14–17).

Workplace violence in healthcare can be exerted by a healthcare user (or family member) or by one or more co-workers. In the literature, the former has been linked to intoxicated individuals (18, 19) and triggered by risk factors such as lack of personnel or poor communication (13). The way this violence is perceived has much to do with its consequences (20). Its impact on professionals ranges from low job satisfaction to somatization, with increased burnout symptoms in the long-term, particularly if it is not physical (21). The somatic alterations observed also act as mediators of anxiety symptomatology in nurses (22). Depression, along with a wide range of emotional disruptions such as anger and fear, has been identified as having a high risk of occurrence in professionals, exposed to threats and violence (23), which is especially problematic in healthcare (24).

Coworker violence is also referred to as lateral violence (LV), horizontal violence, workplace bullying, or incivility (25), terms that often overlap (26). It is a phenomenon of violence between employees with similar ranks and who supposedly hold the same power within the organization, often referred to as coworkers. Its verbal form can be displayed through person-directed attacks (Personal LV), social isolation (Social LV), or work-related violence (Work-related LV) (27).

Nurses are the professionals most affected by LV in healthcare (24, 27). In a 30 years analysis by Rogers (26), it is theorized that nurses interact as members of an oppressed group dominated by medical professionals, leading them to devalue their peers in a passive-aggressive, low-esteem pattern of behavior.

Workplace violence in healthcare in the nursing profession exerted by users is more frequent in mental health services (28–30) and in emergency departments (31, 32), where eight out of ten professionals are subjected to non-physical user violence (UV) (33). On the other hand, LV seems to be more reported in intensive care units and in the emergency departments (34, 35). Operating rooms are also mentioned as danger zones (36, 37).

The adverse impact of LV on nurses has been explored in the literature with alarming conclusions (12, 25), since it is highly correlated with emotional exhaustion, anxiety, somatic symptoms, and depression (27), in addition to poor health (38), lower job satisfaction and high levels of burnout (39). Emotional exhaustion stands out as a major adverse lifelong outcome of LV (40). Furthermore, it has a high positive correlation with intention to change nursing staff, only modulated by job satisfaction (41).

The negative impact of LV on victimized nurses’ health seems to be more damaging than violence coming from third parties, in the sense that users and families are outsiders to the institution and unfamiliar to the victim (38).

Particularly, burnout is a prevalent outcome. It has been divided into three dimensions (42, 43): (a) emotional exhaustion, with the feeling of emptiness as a central element, (b) depersonalization, with a cynical attitude toward work or others, and (c) a low personal sense of professional efficacy, with a generally negative perception of the accomplishments at work, as additional elements. It has been studied not just as an outcome on its own but also as a factor influencing nurses’ job satisfaction and turnover intention (44).

Gender differences may differ if other variables are taken into account, although it has been identified that women are at greater risk of being harassed in the workplace, other variables, such as the setting, may cause men to be at higher risk (27). In the mental health setting, studies point to a greater risk for male nurses, as they are often called upon to restrain agitated patients (30).

Workplace violence in healthcare also has a cost to organizations, such as absenteeism and reduced quality, and it is impossible to rule out that the quality of work and patient care may also be affected (44–46).

### Hypotheses and study goals

Based on previous literature, it is hypothesized that workplace violence (coworkers and UV) perceived by nursing professionals will influence the health of the professionals. Specifically, the greater the exposure to violence, the greater the burnout and the worse the health consequences. In turn, burnout will act as a moderator between the variables coworker violence and health.

To test these hypotheses, the main objective of this study is to explore the relationship between coworker violence (personal, social and occupational), UV (physical and non-physical), general health, and the three classic dimensions of burnout (emotional exhaustion, professional efficacy, and cynicism) using structural equations modeling (SEM).

## Materials and methods

### Participants

The sample used by Pina et al. (40) and Vidal-Alves et al. (27) was used for this study. It consisted of 950 nursing professionals from 13 public hospitals in southeastern Spain, randomly selected by blocking. Of the 13 hospitals, 6 were considered large (200-bed capacity or higher) and 7 were considered medium or small (less than 200-bed capacity). Regarding the characteristics of the sample (Table 1), the age of the participants ranged from 30 to 50 years, with a mean of 39.43 (SD = 9.65). Most were female (77.8%) and had a spouse or life partner (63.2%). Regarding professional characteristics, the mean experience of nursing professionals was 14.02 years and 54.3% had an experience of 5-year or less. In addition, 54% had the same job position for the last 5 years (mean 7.31 years, SD = 8.35).

**TABLE 1:** Correlations.

	1	2	3	4	5	6	7	8	9
1. Emotional exhaustion	1								
2. Professional efficacy	−0.067*	1							
3. Cynicism/Depersonalization	0.504**	−0.109**	1						
4. Total GHQ	0.513**	−0.056*	0.383**	1					
5. Non-physical user violence	0.263**	–0.044	0.232**	0.222**	1				
6. Physical user violence	0.097**	–0.016	0.139**	0.112**	0.402**	1			
7. Co-workers personal violence	0.260**	–0.038	0.228**	0.260**	0.260**	0.155**	1		
8. Co-workers social violence	0.169**	–0.034	0.193**	0.206**	0.206**	0.118**	0.572**	1	
9. Co-workers work-related violence	0.156**	–0.012	0.187**	0.207**	0.207**	0.102**	0.492**	0.545**	1

*p < 0.05; **p < 0.001.

Of the studied sample, 20.3% of the nurses worked in surgery, 17% in internal medicine, 14.3% in the emergency department, 6.9% in nursing, 5.5% in mental health, and 14.8% in other services.

### Design and procedures

A cross-sectional associative design was used. We firstly held a meeting with the boards of directors of the participating hospitals to explain its goals and request approval from their ethics committee. After the approval, the researchers held meetings with each of the ward directors and/or chiefs of staff to request their support in obtaining a representative sample. Once the research team was completed, the study protocol, comprising an informative note, general instructions of the procedure, and the questionnaires, was sent to 50% of the nursing staff (randomly selected). All this information was delivered together with an envelope and the informed consent form, with the recommendation to deliver the completed protocol inside the sealed envelope to the member of the research team responsible for this collection in each hospital. The inclusion criteria for the final sample were: (a) having a current employment contract with the selected center and (b) being a nursing professional or nursing assistant. The applied exclusion criteria were: (a) failure to return the envelope with the filled questionnaires enclosed within the previewed time, (b) not having the informed consent form duly signed, and (c) having less than one month’s work experience in the current center. The response rate was 70.48%.

### Instruments

Besides the sociodemographic and occupational variables, such as age, sex, years of experience in the job/position, type of hospital, and others, we used the following scales assessing different variables, namely UV against healthcare professionals, violence among co-workers, henceforth referred to as LV, burnout, job satisfaction, and general health.

To assess violence between co-workers, the Health Aggressive Behavior Scale – Co-workers and Superiors (HABS-CS) was used (47). It encompasses 10 grouped into three factors: personal factors (3 items), social factors (3 items), and work-related factors (4 items). It is a 10-item Likert-type scale with responses ranging from 1 (never) to 6 (daily). These items are grouped into three factors: personal factors, social factors, and work-related factors, including items such as “Some coworkers play ironic jokes on me” and vertical violence items such as “My superior impairs my participation in training, teaching, or research activities.” The internal consistency observed for this scale was 0.87. The internal consistency observed for this scale was 0.87.

To assess UV against healthcare personnel, the Health Aggressive Behavior Scale – Users was used, a 10-item tool organized into two factors: physical violence (4 items) and non-physical violence (6 items), with responses ranging from 1 = never to 6 = daily. Sentences such as “Users make ironic comments to me” and “Users get angry with me because of delay” where posed. It measures violence from low to medium intensity and obtained an internal consistency of 0.85.

Burnout symptoms were addressed using the Spanish version of Maslach Burnout Inventory – General Survey (MBI-GS) (48). It includes 16 items grouped in three dimensions: emotional exhaustion, professional efficacy, and cynicism, with 5, 5, and 6 items, respectively. This 16-item instrument allows responses ranging from 0 = never to 6 = always, within the six classical dimensions of burnout: cynicism, emotional exhaustion, and professional efficacy. It presents an internal consistency of 0.82.

To assess general health, the validated version of the General Health Questionnaire (GHQ-28) in Spanish was used. This is a 28-item scale to measure the intensity (from 0 to 3) of the symptoms included in the following 4 factors: psychological somatic symptoms (7 items), anxiety and insomnia (7 items), social dysfunction (7 items), and depressive symptoms (7 items). Participants are requested to indicate how their health in general has been over the past few weeks, relying on behavioral items with a 4-point scale indicating the following frequencies of experience: “not at all,” “no more than usual,” “rather more than usual,” and “much more than usual.” Items such as “Lost much sleep over worry” and “Been able to enjoy normal day-to-day activities” are intended to measure recipient’s general health perception. For the present study, the GHQ-28 subscale scores were grouped into a single indicator: General Health. The internal consistency of the measure for this sample was 0.80.

### Data analysis

All descriptive statistics were calculated as well as the relationship between variables using Spearman’s correlation test, to determine whether these variables could be mediators in the model to be tested. A total of four mediation models were extracted: separation of perceived violence (users or co-workers) as predictors of the professionals’ psychological health; using the burnout variables with significant association.

Furthermore, we collected information on each regression coefficient with and without the mediation model, both for direct estimations and standardized ones. To sustain the statistical power, without the need of assuming multivariant normality of the distributions of each sample, a bootstrap analysis was conducted for estimating the indirect effects of the mediations (49, 50).

Structural equation analysis is a multivariate inferential technique that tests causal models with both indicators, which are observable variables, and latent variables (non-observable). The use of Structural Equation Modeling (SEM) consists of the analysis of covariance structures, and simultaneous equations modeling (causal modeling). This technique allows an approach to hypothesis testing by model confirmation (51).

**FIGURE 1:**
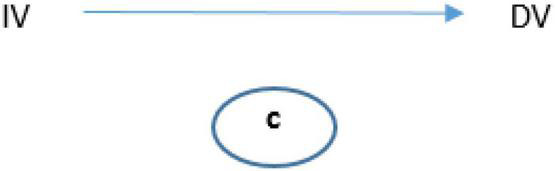
Basic mediation model used.

**FIGURE 2:**
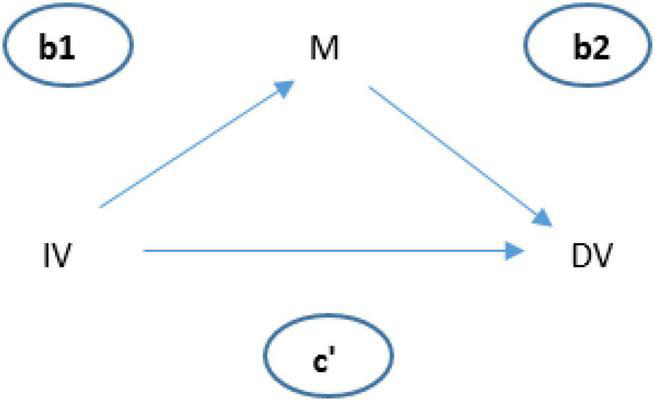
Relationship between IV and DV.

The mediation model tests whether c is significantly distinct from c′ (Figure 1) to determine that the relationship between the independent variable (IV) and dependent variable (DV) is indirectly explained (Figure 2).

Structural equations modeling is used to test the models with indicators (observable variables) and latent variables (non-observable). It allows testing and confirming the model, with high accuracy and allows testing the theoretical model of the relationship between a set of indicators (path analysis). To avoid measurement error (such as an exogenous latent variable), a set of observable variables was addressed as indicators of the impact of a latent variable. Thus, a latent variable with several indicators and SEM were used to test our causal model. The steps were: (a) model formulation (measurement model and structural model included); (b) model identification; (c) model estimation; (d) model evaluation; and (e) model modification.

## Results

From the correlation study, it could be extracted that there is a positive correlation between the variable of Emotional Exhaustion and: Depersonalization (*r* = 0.504, *p* < 0.01), General Health (*r* = 0.513, *p* < 0.01), Non-physical UV (*r* = 0.263, *p* < 0.01), physical UV (*r* = 0.097, *p* < 0.01), and Personal LV (*r* = 0.260, *p* < 0.01), Social LV (*r* = 0.169, *p* < 0.01), and Work-related LV (*r* = 0.156, *p* < 0.01). The professional efficacy variable only demonstrated a correlation with Depersonalization (*r* = −0.109, *p* < 0.01) and General Health (*r* = −0.056, *p* > 0.05), although the latter is a small size correlation.

Regarding Depersonalization, this variable presented a significant correlation with General Health (*r* = 0.383, *p* < 0.01), Non-physical UV (*r* = 0.232, *p* < 0.01), Physical UV (*r* = 0.139, *p* < 0.01) and Personal LV (*r* = 0.228^**^, *p* < 0.01), Social LV (*r* = 0.193, *p* < 0.01), and Work-related LV (*r* = 0.187, *p* < 0.01). The other variables inter-correlated positively and significantly within a range from 0.112 and 0.545 (*p* < 0.01). Considering the correlations obtained, the Emotional Exhaustion and Cynicism/Depersonalization variables were tested as mediators between perceived violence (both by users and co-workers) and General Health, measured with the GHQ-28.

**FIGURE 3:**
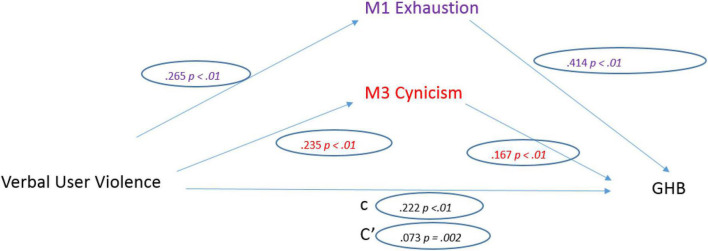
Non-physical user violence model.

Firstly, the model was tested by considering the observed scores on Non-physical UV against health professionals (Figure 1). Non-physical UV was a significant predictor of General Health (*b* = 0.09, SE = 0.011, *p* < 0.001). The standardized coefficient of 0.222 reflects the direct effect of Non-physical UV on General Health, path “c” in the model (Figures 1, 3).

Regarding the three mediators, non-physical UV was a significant predictor of Emotional Exhaustion (*b* = 0.228, SE = 0.022, *p* < 0.001). The standardized regression coefficient for this path is 0.265. Non-physical UV was a significant predictor of Depersonalization (*b* = 0.189, SE = 0.021, *p* < 0.001). The standardized regression coefficient for this path was 0.235.

After controlling the effects of the mediators, Non-physical UV was a significant predictor of General Health (*b* = 0.03, SE = 0.009, *p* = 0.002). The standardized regression coefficient for this path is 0.073. The total indirect effect was significant (0.061, CI [0.046, 0.077]).

As for physical UV, it was a significant predictor of General Health (*b* = 0.230, SE = 0.053, *p* < 0.001). The standardized coefficient of 0.113 reflects the direct effect of Physical UV on General Health, the “c” path of the model.

**FIGURE 4:**
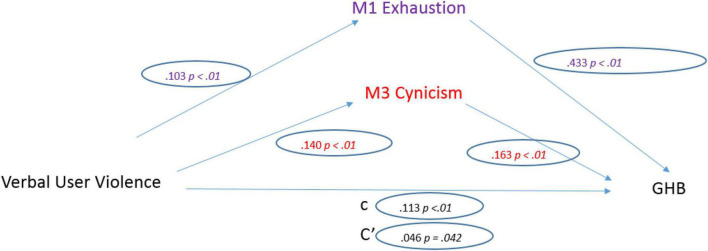
Physical user violence model.

Regarding the three mediators, Physical UV was a significant predictor of Emotional Exhaustion (*b* = 0.442, SE = 0.112, *p* < 0.001). The standardized regression coefficient for this path is 0.265. Physical UV was a significant predictor of Depersonalization (*b* = 0.565, SE = 0.105, *p* < 0.001). The standardized regression coefficient for this path was 0.140 (Figure 4).

After controlling the effects of the mediators, Physical UV was a significant predictor of General Health (*b* = 0.093, SE = 0.046, *p* = 0.042). The standardized regression coefficient for this path is 0.046. The total indirect effect was significant (0.138, CI [0.067, 0.213]).

**FIGURE 5:**
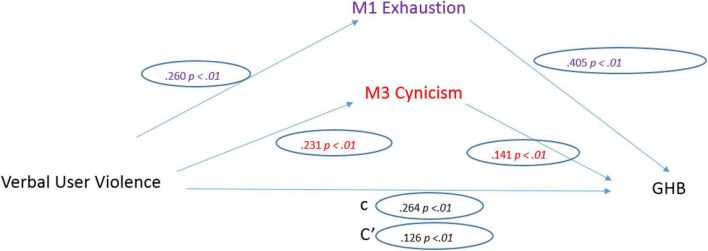
Personal co-worker violence model.

When analyzing co-worker violence, the Personal-related LV factor emerged as a significant predictor of General Health (*b* = 0.214, SE = 0.021, *p* < 0.001). The standardized coefficient of 0.264 reflects the direct effect of Personal-related LV on General Health, the “c” path in the model (Figure 5).

Regarding the three mediators, personal-related LV was a significant predictor of Emotional Exhaustion (*b* = 0.449, SE = 0.045, *p* < 0.001). The standardized regression coefficient for this path is 0.260. Personal-related LV was a significant predictor of Depersonalization (*b* = 0.375, SE = 0.042, *p* < 0.001). The standardized regression coefficient for this path was 0.231.

Once the effects of the mediators were controlled, Personal-related LV was a significant predictor of General Health (*b* = 0.102, SE = 0.019, *p* < 0.001). The standardized regression coefficient for this path is 0.126. The total indirect effect was significant (0.112, CI [0.086, 0.140]).

**FIGURE 6:**
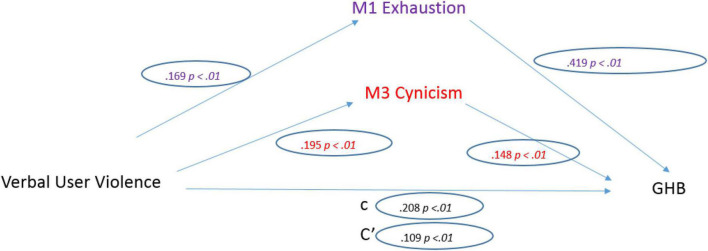
Social co-worker violence model.

Comparatively, social co-worker violence was a significant predictor of General Health (*b* = 0.272, SE = 0.034, *p* < 0.001). The standardized coefficient of 0.208 reflects the direct effect of Social LV on General Health, the “c” path in the model (Figure 6).

Regarding the three mediators: Social LV was a significant predictor of Emotional Exhaustion (*b* = 0.469, SE = 0.072, *p* < 0.001). The standardized regression coefficient for this path is 0.169. Social LV was a significant predictor of Depersonalization (*b* = 0.509, SE = 0.067, *p* < 0.001). The standardized regression coefficient for this path was 0.195.

After controlling the effects of the mediators, Social LV was a significant predictor of General Health (*b* = 0.142, SE = 0.012, *p* < 0.001). The standardized regression coefficient for this path is 0.109. The total indirect effect was significant (0.130, CI [0.085, 0.180]).

Finally, work-related co-worker violence was a significant predictor of General Health (*b* = 0.359, SE = 0.044, *p* < 0.001). The standardized coefficient of 0.212 reflects the direct effect of Work-related LV on GHB, the “c” path in the model.

**FIGURE 7:**
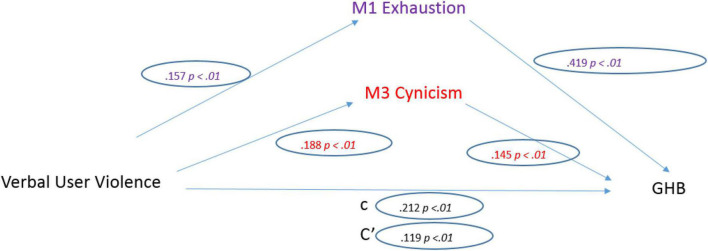
Work-related co-worker violence model.

Regarding the three mediators: Work-related LV was a significant predictor of Emotional Exhaustion (*b* = 0.563, SE = 0.094, *p* < 0.001). The standardized regression coefficient for this path is 0.157. Work-related LV was a significant predictor of Depersonalization (*b* = 0.637, SE = 0.088, *p* < 0.001). The standardized regression coefficient for this path was 0.188 (Figure 7).

After controlling the effects of the mediators, Work-related LV was a significant predictor of General Health (*b* = 0.201, SE = 0.038, *p* < 0.001). The standardized regression coefficient for this path is 0.119. The total indirect effect was significant (0.158, CI [0.103, 0.222]).

## Discussion

The results presented support the hypothesized model. Exposure to WPV leads to poorer perceived health among nurses when mediated by burnout, especially emotional exhaustion, adding evidence to findings that relate more WPV to more burnout (52).

In our study, the negative impact of WPVH is highlighted by the strong positive correlations observed between the three subtypes of LV and both subtypes of UV and decreased quality of health indicators. Preventing stressors such as WPV is a step to improve work environment (44) and a key element to improve nurses’ health and prevent decreased work quality or turnover intent (53).

Burnout, as a psychological stress response to demanding work conditions, usually begins with symptoms of emotional exhaustion (42). Described as a feeling of emptiness and exhaustion as a result of high levels of stress (42), this central feature of burnout appears in our results as a particularly important adverse outcome of the subtypes of both UV and LV that can be seen as more subtle: Non-physical UV and Personal LV, respectively. This piece of evidence corroborates the powerful negative effect of latent forms of aggression, that is not easily noticed by the victim or bystanders (54).

Additionally, it is a significant predictor of poorer health perception. Figure 3 shows an indirect relationship between UV and the nurses’ general perceived health, with Emotional Exhaustion and cynicism as mediators. Verbal violence causes high levels of these two dimensions of burnout, which, in turn, generates a deterioration in general health.

This is a dangerous aspect of WPVH, since, as it is not easily identified, it allows the problem to persist and insidiously destroy the victim’s ability to function in a medium and long-term (12, 21, 55). Verbal and psychological abuse is actually the most frequently observed in health institutions (13). This refers to a latent form of violence (1), based on person-directed abuse, using manipulation, misleading, or lying to disrupt the victim (2, 7) and, as described by Griffin (56), “non-verbal innuendo (raising eyebrows, making a face)” and “verbal affront (covering up, snide remarks, lack of openness, and abrupt responses).”

In this context, Depersonalization, with its cynical component, emerges as a subsequent response to work-related stress and is related to the interpersonal dimension of burnout (57). This detachment response is the result of the overload of emotional exhaustion and loss of idealism, leading the professional to shift from giving their maximum efforts to the job to giving their bare minimum (58). It is often used by professionals to protect themselves from especially harmful work-related aspects at first, but results in severe dehumanization (42, 58–60).

Consistent with previous studies, Depersonalization is overall positively correlated to UV dimensions and LV (61, 62). Considering its reported impact on nurses’ turnover intentions (53, 63), and also on decision-making and patient care itself (64), our findings suggest that nurses would benefit from direct action to prevent violence from occurring, namely improving work relations (44).

### User violence explaining models

Nurses’ accounts of UV can be overwhelming and help explain how toxic levels of stress related to dealing with violent patients and their relatives prepare the ground for high levels of suffering, with impacts on mental and physical health (65).

Tested models focusing on the different subtypes of violence show how strong the relationships between perceived UV subtypes and General Health, especially with the mediating role of Emotional Exhaustion.

### Non-physical user violence

Using Depersonalization and Emotional Exhaustion as mediators, our SEM gives a rough indication of the indirect effect of verbal (non-physical) UV and poorer general health outcomes through burnout, as a cumulative effect of stress. Our results add evidence to those of other authors on the widespread exposure of nurses to user hostility in specific healthcare services (14, 16, 32, 39) and the role of Emotional Exhaustion, either as a burden to the professionals’ wellbeing (21) or as an obstacle to professional efficacy (32). From jocular comments to life threats, nurses face daily non-physical aggressions that concur with somatic symptoms, anxiety, social dysfunction, and depression (32), which may be more impactful if nurses are not supported or motivated (39).

### Physical user violence

The current results also present a high prevalence of UV Physical violence as was observed by Li et al. (66) and a high impact of Physical Violence on general health found by other studies (13, 24, 67) with and without the two mentioned dimensions of burnout as mediators, but stronger when both are used. This is supported by other studies acknowledging that burnout is correlated to physical injuries and specific diseases (68) but, concurrently, mediates depressive symptoms and low physical energy levels, as a result of WPVH (55). Physical violence in hospitals includes severe episodes of kicking, punching, and use of cold weapons, especially toward male nurses by patients’ relatives, and when the patient is perceived by the aggressor as more vulnerable (69).

In fact, the model further depicts this form of violence by users as a strong predictor of Emotional Exhaustion and Depersonalization, which emphasizes the urgency of preventing aggression because of its clear pathway to burnout highlighted by other authors (70). Interestingly, it also has an impact on general health, but at a less significant level, which was also pointed out by Volz et al. (64).

### Lateral violence explaining models

Our LV results point to Personal LV having a direct negative effect on victims’ health and, regardless of mediators, but shows Depersonalization and particularly Emotional Exhaustion, as strong predictors. Our data points to a direct pathway between the perception of personal attacks (verbal and non-verbal) by coworkers and the health problems of the affected professionals.

Social LV lies in the obstacles posed to the victim in reaching information and getting ostracized, humiliated, or ridiculed (40, 71). Workplaces are often a place where nurses’ narratives go beyond the loss of idealism and reflect the realization of the pervasiveness of a culture of violence (25). Our SEM particularly indicates that Emotional Exhaustion is strongly predicted by this social type of LV that encompasses harassment behaviors, often based on ganging up against a coworker who is intimidated and isolated. But, more expressively, it evinces its mediating role toward nurses’ General Health, notwithstanding, the direct impact of Social LV on health, which is consistently referred to in observations of workplace culture as acts of social exclusion, using silence, speaking in the back and personal derogation (35, 47, 72, 73). Burnout is described as an accumulated emotion (44), and its reduction is possible through the use of violence prevention strategies by hospital managers to improve nurses’ health perception and preserve their nursing workforce.

Our results do not support that violence exerted by a coworker is more impactful, long-lasting, and overall deleterious to nurses’ physical and mental health than external violence, exerted by users and their relatives (38), since our SEM did not depict higher effects of LV directly on health or through burnout pathways.

The culture of WPVH produces cases in which nurses are victims, but also perpetrators (35), raising concerns about the ubiquity of favorable attitudes toward this sort of violence in nursing. The attacks on coworkers are often focused on undermining colleagues to get ahead and gain recognition in the workplace, regardless of ethical concerns (25, 69). Work-related LV is often used to exert power and destroy self-esteem, especially in those less powerful, and generates feelings of suspicion and lack of trust (25, 26, 73). The theory of the Oppressed Group refers to the insecurity of perpetrators (about their abilities or other aspects of the self) as a risk factor of LV (26) employed in the rite of passage of nursing work pathways (73). This may be self-defensive, in the face of high job demands, but also well-intentioned when it emerges as a learned tough love approach that is conveyed to every newly graduated nurse so that they are prepared for the nursing profession (27, 74).

Consistently, our Work-related LV model adds evidence to its direct relation to Health and to the highly significant modulating role of Emotional Exhaustion.

The awareness of professionals is so fundamental when evidence shows that victims do not always acknowledge abusive behaviors as such and perpetrators do not always foresee the impact of their actions until it is too late for many nurses.

As the first sign of burnout, Emotional Exhaustion requires an immediate organizational response (73, 74), but an appropriate prevention action that tackles attitudes toward nursing education in higher degree institutions in the first place, can prevent the early signs and promote a healthy workplace environment.

## Conclusion

The present findings support the hypothesized model and reinforce the conclusions of previous studies. They explain the mediation role of burnout in health deterioration as a cumulative effect of exposure to violence, either from users or co-workers. Nurses are at great health risk for they high exposure to workplace violence combined with enduring it for long periods, concurring to burnout indicators such as detachment and emotional exhaustion.

They suggest the benefit of benchmarking actions against violence in the workplace, both to provide training on improving communication skills. Valuable actions against LV would include a new training framework in nursing based in skill-building that tackles communication skills, empathy building, and awareness about what is and what is not acceptable behavior, besides an adequate organizational response to victims that allows them to come forward, without fear of consequences.

Future research might profit from bringing attention to nursing education and training to change from the current sink or swim mindset to a more empathic one. Also addressing bystander awareness and empowerment since it may be an important pathway to encourage early detection of violence, victims support, and an overall improvement of work environment.

## Limitations

The interpretation of our results must take into consideration the following limitations. The use of self-report measures is frequently accompanied by bias. Other variables of interest have not been taken into account in the models explored, which is a limitation of the models. Future studies could benefit from considering objective indicators, such as rates of medical leave of nursing personnel.

## Data availability statement

The raw data supporting the conclusions of this article will be made available by the authors, without undue reservation.

## Ethics statement

The studies involving human participants were reviewed and approved by the Research Ethics Committee of the authors’ home university (ID: 3555/2021). The patients/participants provided their written informed consent to participate in this study.

## Author contributions

DPi and JR-H: conceptualization. EP-L: methodology and investigation. DPa: formal analysis. MV-A: writing—original draft preparation. DPi, JR-H, and BM-J: writing—review and editing. BM-J: funding acquisition. All authors have read and agreed to the published version of the manuscript.
